# Community and Hospital Factors Associated With Stroke Center Certification in the United States, 2009 to 2017

**DOI:** 10.1001/jamanetworkopen.2019.7855

**Published:** 2019-07-26

**Authors:** Yu-Chu Shen, Gabriel Chen, Renee Y. Hsia

**Affiliations:** 1 Graduate School of Business and Public Policy, Naval Postgraduate School, Monterey, California; 2 National Bureau of Economic Research, Cambridge, Massachusetts; 3 Ministry of Defense, Singapore; 4 Department of Emergency Medicine, University of California at San Francisco; 5 Philip R. Lee Institute for Health Policy Studies, University of California at San Francisco

## Abstract

**Importance:**

The increased number of stroke centers in the United States may not be equitably distributed across all populations. Anecdotal reports suggest there may be differential proliferation in wealthier and urban communities.

**Objective:**

To examine hospital characteristics and economic conditions of communities surrounding hospitals with and without stroke centers.

**Design, Setting, and Participants:**

This cohort study included all general, short-term, acute hospitals in the continental United States and used merged data from the Joint Commission, Det Norske Veritas, Healthcare Facilities Accreditation Program, state health departments, the Centers for Medicare & Medicaid Services, the American Hospital Association, the Dartmouth Atlas of Health Care, and the US Census Bureau from January 1, 2009, to September 30, 2017, to compare hospital and community characteristics of stroke-certified and non–stroke-certified hospitals and assessed characteristics of early and late adopters of stroke certification.

**Main Outcomes and Measures:**

Stroke center certification was the primary outcome. Risk factors were grouped into 3 categories: economic and financial, hospital, and community characteristics. Survival analyses were performed using a Cox proportional hazards regression model.

**Results:**

The study included 4546 US hospitals. During the study period, 1689 hospitals (37.2%) were stroke certified (961 adopted certification on or before January 1, 2009, 728 afterward). After controlling for other area and hospital characteristics, hospitals in low-income hospital service areas and the lower tertile of profit-margin distribution were less likely to adopt stroke certification (hazard ratio [HR], 0.62; 95% CI, 0.52-0.74 and HR, 0.87; 95% CI, 0.78-0.98, respectively). Urban hospitals had a higher likelihood of stroke certification than rural hospitals (HR, 12.79; 95% CI, 10.64-15.37).

**Conclusions and Relevance:**

This study found that stroke centers have proliferated unevenly across geographic localities, where hospitals in high-income hospital service areas and with higher profit margins have a greater likelihood of being stroke certified. These findings suggest that market-driven factors may be associated with stroke center certification.

## Key Points

**Question:**

What economic and financial, hospital, and community characteristics are associated with stroke center certification of hospitals?

**Findings:**

In this cohort study of 4546 US hospitals, 37% were stroke certified between 2009 and 2017. After controlling for other area and hospital characteristics, hospitals in low-income hospital service areas and in the lower tertile of profit-margin distribution were less likely to adopt stroke certification.

**Meaning:**

These findings suggest that market-driven factors may be associated with stroke center certification.

## Teaser

This cohort study analyzes economic, community, and hospital factors associated with the adoption of stroke center certification for US hospitals from 2009 to 2017.

## Introduction

As the fifth leading cause of death and the leading cause of long-term disability in the United States, stroke has been a health care priority in the United States across the past 2 decades. In June 2000, the Brain Attack Coalition recommended the establishment of primary stroke centers.^1^ In 2003, the Joint Commission, along with the American Heart Association and American Stroke Association, introduced the Primary Stroke Center (PSC) certification to encourage and standardize stroke certification for hospitals.^2^ Since then, other organizations also provide stroke certification. Numerous early studies showed that, compared with non–stroke unit care, organized stroke unit care reduced the risk of death in patients with stroke by 14%, decreased the risk of death or institutionalized care of patients with stroke by 18%, and reduced the risk of death or dependency among patients with stroke by 18%.^3^ Although for many years there was substantial concern that a considerable proportion of the population lacked adequate access to stroke centers,^4,5,6,7^ the number of stroke centers has increased during the past decade.^8^

Because stroke certification is a voluntary program, however, a key concern is that the addition of new stroke centers may be concentrated in certain communities—namely, affluent and urban neighborhoods. If profit-driven factors, rather than the need for stroke care, have contributed to this increase in stroke center certification, this distribution could represent an inefficiency in the allocation of resources for stroke on a systems level, with implications for population-level disparities and also the regionalization of care.

Previous studies on disparities in access to stroke centers have mainly focused on geographic disparities in access to stroke care,^9,10^ including a particular focus on rural-urban disparities,^11,12^ or have examined only racial and income disparities for patients who already receive care within stroke centers^13,14,15,16,17^ or already have presented for care.^18,19,20,21,22^ However, little is known about how hospital characteristics and the population characteristics of communities surrounding hospitals with and without stroke certification programs may differ on a national scale and how these characteristics have changed over time. Our research addresses the factors associated with stroke certification of hospitals, including economic and financial, hospital, and community characteristics, and analyzes these differences between early and late adopters of stroke center status.

## Methods

### Study Design and Data Sources

Our study followed the Strengthening the Reporting of Observational Studies in Epidemiology (STROBE) reporting guideline. We analyzed all general short-term, acute hospitals in the continental United States from January 1, 2009, to September 30, 2017. Hospitals can obtain stroke center certification from the following organizations: the Joint Commission, which is the largest national program, accounting for more than 95% of all national accreditation; Det Norske Veritas–Germanischer Lloyd (DNV GL) (formerly DNV); the Healthcare Facilities Accreditation Program (HFAP) (also known as AOA [American Osteopathic Association]/HFAP); and state bodies. Some hospitals carry certifications from multiple agencies. We identified stroke designation status using the following sources. First, we obtained a list of hospitals and the year and quarter that they were certified by the Joint Commission. Second, we supplemented the Joint Commission data with a list of other stroke-certified hospitals and the year that they were certified by DNV GL, HFAP, and state health departments between January 1, 2009, and December 31, 2013, which was shared by the Uchino et al group.^23^ Based on patterns observed in the Joint Commission data, we updated the supplemental data to September 30, 2017, assuming hospitals did not lose certification from these programs once they adopted the status. Our sample captures 4546 hospitals, of which 1416 were certified by the Joint Commission (31.1% of the hospital universe). Two hundred twenty-six hospitals (5.0%) were certified by state bodies without Joint Commission designation, and the remaining 47 hospitals (1.0%) were certified by the other 2 national programs without Joint Commission designation. This study was deemed exempt from review by the institutional review board at the University of California, San Francisco.

We obtained hospital organizational information from the American Hospital Association Annual Survey, such as ownership types, available technological capabilities, number of hospital beds, teaching status, and whether a hospital is a member of a system. We obtained financial and additional organizational information from the Centers for Medicare & Medicaid Services Healthcare Cost Report Information System, including cost and revenue and case mix index. We also captured economic and demographic characteristics of communities at the zip code level from the 2010 US Census and additionally grouped the zip code communities by the hospital service area (HSA) level using a crosswalk from the Dartmouth Atlas of Health Care.^24^

### Outcome Measure

Stroke certification was the primary outcome in our analysis. To date, stroke certification programs have continued to evolve across all accreditation bodies and mainly include 3 core certifications based on diagnostic testing, neurosurgical services, and clinical performance standards. Ranking from least to greatest capabilities, these include Acute Stroke Ready Hospitals (introduced by the Joint Commission in 2015), PSC (the only designation introduced by the Joint Commission in 2003), and Comprehensive Stroke Center (introduced by the Joint Commission in 2010). In addition, the Joint Commission introduced the Thrombectomy-Capable Stroke Center in 2018, and HFAP is considering a similar categorization (Thrombectomy Proficient).^25^ As done in previous literature,^23^ we coded stroke certification as binary to capture hospitals that have adopted at least PSC level from at least 1 of the 4 certification programs. Furthermore, we classified hospitals as early adopters if they were certified within the first 6 years of the PSC program implementation (ie, December 31, 2009, or before) and late adopters if certified on or after January 1, 2010.

### Explanatory Variables

We group the risk factors into 3 categories: economic and financial characteristics, hospital characteristics, and community characteristics. We defined the community a hospital serves using the HSA definition developed by the Dartmouth Atlas of Health Care.^24^ For a given hospital, its HSA includes population from a set of zip codes whose residents received most of their hospitalizations from that hospital.

#### Economic and Financial Characteristics

To investigate whether there are systematic differences in stroke certification adoption rate across hospitals that serve communities of different income levels, we grouped each hospital’s HSA into low-, middle-, and high-income communities based on the tertiles of the empirical distribution of median family income from the 2010 US Census. To investigate whether adoption rate differs across hospitals of different financial conditions, we included hospital profit margin, defined as the ratio of net revenue to total operating costs. For ease of interpretation and to capture any possible nonlinear association, we further divided hospitals into 3 categories based on the tertiles of the profit-margin distribution.

#### Hospital Characteristics

We analyzed hospital characteristics by ownership type (not for profit, for profit, and government); teaching status (1 if the hospital has a resident to bed ratio >0.25 or has a medical school affiliation; otherwise, 0); systems membership; critical access hospital status; hospital size (in the following categories by total hospital beds: <100 [reference group], 100-399, and ≥400); and availability of high-cost technical capabilities, proxied by whether a hospital has either a percutaneous coronary intervention laboratory or the capability to perform coronary artery bypass graft. In addition, we included hospitals’ case mix index value as a proxy for general underlying sickness of the patient population.

#### Community Characteristics

We included 2 geographic characteristics—whether a hospital is located in a “stroke belt” state and whether it is located in an urban area. Stroke belt states are defined as a group of 11 states in the Southeastern United States with historically higher than average stroke mortality.^26^ Urban hospitals were identified from the Healthcare Cost Report Information System. We also included a market measure that takes on the value of 1 if there is another stroke center within a 24-km radius (equivalent to 15 miles) of the hospital’s location. In addition, we included each HSA’s population size (log transformed) and the percentage of HSA population who are older than 65 years.

### Statistical Analysis

We analyzed the association between stroke certification adoption rate and hospital and community characteristics using the Cox proportional hazards regression model.^27^ The model estimated a hazard function representing the probability of a hospital obtaining stroke certification in quarter *t*, conditional on the hospital not being stroke certified up to the start of that period (*t*). A hospital entered the risk window of the empirical model in January 1, 2009, or the first quarter it appeared in the data. A hospital exited the model after the quarter it became stroke certified or after the last observation (ie, right-censored) if it did not adopt stroke center certification.

Because it is impossible to have 2 completely orthogonal variables when analyzing hospital factors, we chose our variables based on what earlier literature and our conceptual framework dictated would be important factors to consider. We began with a set of bivariate models, wherein we implemented the Cox model between each risk factor and stroke certification. The bivariate model allowed us to estimate the overall effect of each risk factor. We then implemented a multivariate analysis that included the complete set of risk factors, which allowed us to estimate the association between a hospital’s decision to adopt stroke certification and a single risk factor, assuming we compared hospitals that were comparable in all other dimensions. In other words, the multivariate model had the important function of allowing us to ask the hypothetical question: if 2 hospitals are identical in all other dimensions, what are their differences in adopting stroke certification if they differ in only 1 dimension?

We also performed a sensitivity analysis that used the share of the HSA population below the federal poverty line as an alternate measure of community economic condition. Because our community variables were measured at the HSA level, whereby some HSAs contained multiple hospitals (40% of HSAs contained >1 hospital), we estimated robust SEs that took into account clustering of hospitals at the HSA level for all models.

We performed proportionality tests for all variables using the standard 5% significance level to determine statistical significance (2 of these variables, cardiac capability and system membership, failed the proportional hazards test). We report Bonferroni-adjusted *P* value using 2-sided comparisons.^28^ We also repeated the survival model separately for hospitals operating in rural communities from those in urban communities to investigate further whether factors that influence a hospital’s decision to adopt stroke center certification differ between hospitals operating in rural and urban areas. We performed all analyses using Stata software, version 15 (StataCorp).

## Results

**Figure 1.**
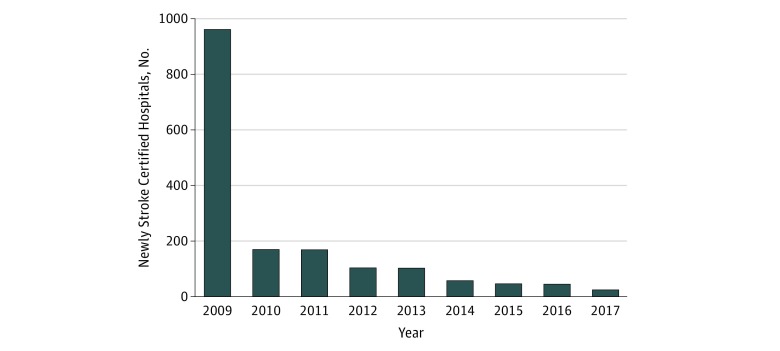
Number of Stroke-Certified Hospitals Annually Data for 2009 represent all hospitals that were stroke certified on or before December 31, 2009.

**Figure 2.**
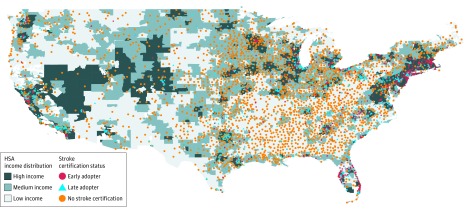
Stroke-Certified Hospital Locations and Hospital Service Area (HSA) Income Distribution As seen on the map, stroke-certified hospitals generally appear in high-income (ie, darker-shaded) areas, with few exceptions.

We studied a total of 4546 unique hospitals, with 104 079 hospital-quarter observations. As of July 1, 2017, 1689 hospitals (37.2%) in the United States were stroke certified. Among the 1689 stroke centers, 961 were certified as of 2009 (ie, early adopters) and 728 were late adopters (Figure 1). Figure 2 illustrates the location of stroke-certified hospitals and non–stroke-certified hospitals and income levels of the HSA. As seen on the map, the stroke-certified hospitals generally appear in high-income areas, with a few exceptions.

### Stroke-Certified vs Non–Stroke-Certified Hospitals

Table 1 provides data showing significant differences in economic and financial characteristics between stroke-certified and non–stroke-certified hospitals. Non–stroke-certified hospitals served communities with a mean (SD) family income of $55 428 ($13 487), compared with $70 633 ($20 301) for stroke-certified hospitals. Stroke-certified hospitals did better financially, with a mean (SD) profit margin of 0.02 (0.21) for stroke-certified hospitals and −0.02 (0.15) for non–stroke-certified hospitals. Stroke-certified hospitals were more likely than non–stroke-certified hospitals to have better hospital resources and capabilities: 1357 stroke-certified hospitals (80.3%) had cardiac capacity vs 563 non–stroke-certified hospitals (19.7%). Stroke-certified hospitals also tended to be larger than non–stroke-certified hospitals (164 stroke-certified hospitals [9.7%] and 2034 non–stroke-certified hospitals [71.2%] operate <100 beds). Stroke-certified hospitals were more likely than non–stroke-certified hospitals to be located in urban communities (1561 [92.4%] vs 1055 [36.9%]; *P* < .001) and less likely to be located in stroke belt states (196 [11.6%] vs 616 [21.6%]; *P* < .001).

**Table 1.** Baseline Hospital and Area Characteristics of 4546 US Hospitals Overall and by Stroke Certification Status, 2009-2017

Characteristic	No. (%)				
	All Hospitals (N = 4546)^a^	Non–Stroke-Certified Hospitals (n = 2857)	All Certified Hospitals (n = 1689)	Early Adopters (n = 961)^b^	Late Adopters (n = 728)
**Economic and Financial Characteristics**					
HAS annual family income, mean (SD), $	61 077 (17 927)	55 428 (13 487)	70 633 (20 301)	74 495 (21 925)	65 535 (16 627)
Profit margin, mean (SD)^c^	−0.01 (0.18)	−0.02 (0.15)	0.02 (0.21)	0.02 (0.24)	0.02 (0.16)
**Hospital Characteristics**					
Ownership					
Not for profit	2727 (60.0)	1499 (52.5)	1228 (72.7)	742 (77.2)	486 (66.8)
For profit	797 (17.5)	515 (18.0)	282 (16.7)	127 (13.2)	155 (21.3)
Government	1005 (22.1)	827 (28.9)	178 (10.5)	92 (9.6)	86 (11.8)
Critical access hospital	1139 (25.0)	1126 (39.4)	13 (0.8)	4 (0.4)	9 (1.2)
Total beds					
<100	2198 (48.3)	2034 (71.2)	164 (9.7)	45 (4.7)	119 (16.3)
100-399	1894 (41.7)	791 (27.7)	1103 (65.3)	587 (61.1)	516 (70.9)
≥400	454 (10.0)	32 (1.1)	422 (25.0)	329 (34.2)	93 (12.8)
Teaching hospital	1022 (22.5)	256 (9.0)	766 (45.3)	539 (56.1)	227 (31.2)
Cardiac capacity (PCI laboratory or CABG)	1920 (42.2)	563 (19.7)	1357 (80.3)	811 (84.4)	546 (75.0)
Member of a hospital system	2553 (56.2)	1366 (47.8)	1187 (70.3)	678 (70.6)	509 (69.9)
Case mix index, mean (SD)	1.38 (24)	1.31 (22)	1.50 (22)	1.54 (22)	1.44 (20)
**Community Characteristics**					
Stroke belt states	812 (17.9)	616 (21.6)	196 (11.6)	84 (8.7)	112 (15.4)
Another stroke center within 24-km radius	686 (15.1)	183 (6.4)	503 (30.0)	325 (33.8)	178 (24.4)
Urban	2616 (57.5)	1055 (36.9)	1561 (92.4)	927 (96.5)	634 (87.1)
Total population in HSA, mean (SD)	31 2287 (587 055)	191 493 (483 872)	516 613 (682 151)	559 504 (676 619)	459 995 (685 751)
Percentage of population in HSA aged ≥65 y, mean (SD)	14.67 (4.28)	15.44 (4.16)	13.39 (4.18)	13.69 (4.34)	12.99 (3.94)

Abbreviations: CABG, coronary artery bypass graft; HSA, hospital service areas; PCI, percutaneous coronary intervention.

^a^ Refers to the total number of unique hospitals studied between 2009 to 2017. Depending on the availability of records and the individual hospital’s time of inception, the number of years tracked may differ from hospital to hospital.

^b^ Includes all hospitals stroke certified on or before December 31, 2009.

^c^ Profit margin is computed as the difference of net revenue minus total operating expenditure divided by total operating expenditure.

### Early vs Late Adopters

Table 1 reveals systematic differences between early and late adopters of stroke certification. Stroke centers appeared primarily in high-income communities in the beginning; mean (SD) family income in communities was $74 495 ($21 925) for early adopters and $65 535 ($16 627) for late adopters. Early adopters were more likely than late adopters to have cardiac capacity (811 of 961 [84.4%] vs 546 of 728 [75.0%]), manage more complex cases (mean case mix index, 1.54 vs 1.44), and were larger in size.

### Bivariate Analyses

**Figure 3.**
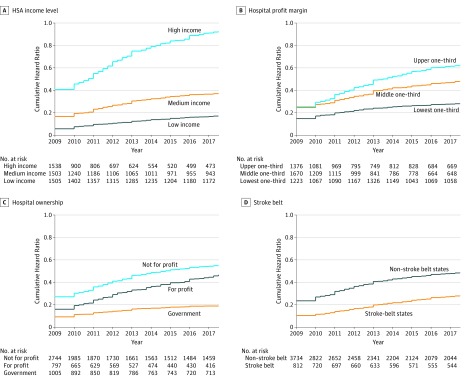
Cumulative Hazard Curves of Adopting Stroke Certification of All Hospitals, by Hospital Characteristics

In bivariate analysis (Table 2), hospitals in low-income and middle-income HSAs were less likely to adopt stroke certification compared with hospitals in high-income HSAs (low-income vs high-income: hazard ratio [HR], 0.1718; 95% CI, 0.1511-0.1954; and middle-income vs high-income: HR, 0.40; 95% CI, 0.35-0.46). Figure 3 shows the corresponding cumulative hazard rate. Hospitals serving high-income communities had a cumulative hazard rate of 0.9259 (95% CI, 0.8641-0.9921) and those serving low-income communities had a cumulative hazard rate 0.17 of adopting stroke certification. Similarly, hospitals in the lower and middle tertiles of profit-margin distribution were less likely to adopt certification (lower tertile: HR, 0.46; 95% CI, 0.41-0.53 and middle tertile: HR, 0.80; 95% CI, 0.72-0.89) than hospitals in the upper tertile, with a cumulative hazard rate of 0.62 (95% CI, 0.58-0.68) for the upper tertile and 0.28 for the lower tertile (95% CI, 0.25-0.31).

**Table 2.** Bivariate and Multivariate HRs of US Hospitals Adopting Stroke Certification, 2009-2017

Risk Factor	Bivariate Model, HR (95% CI)	*P* Value^a^	Multivariate Model, HR (95% CI)	*P* Value^a^
**Economic and Financial Characteristics**				
HSA family income, tertile				
Upper	1 [Reference]			
Middle	0.40 (0.35-0.46)^b^	<.001	0.78 (0.69-0.87)^b^	<.001
Lower	0.18 (0.15-0.21)^b^	<.001	0.62 (0.52-0.74)^b^	<.001
Profit margin, tertile				
Upper	1 [Reference]			
Middle	0.80 (0.72-0.89)^b^	<.001	0.96 (0.87-1.05)	.69
Lower	0.46 (0.41-0.53)^b^	<.001	0.87 (0.78-0.98)^c^	.04
**Hospital Characteristics**				
Ownership				
Not for profit	1 [Reference]			
For profit	0.79 (0.69-0.89)^b^	<.001	0.88 (0.79-0.99)^c^	.07
Government	0.34 (0.29-0.40)^b^	<.001	0.83 (0.72-0.97)^c^	.04
Designated critical access hospital	0.02 (0.01-0.03)^b^	<.001	0.12 (0.07-0.22)^b^	<.001
Total hospital beds				
<100	1 [Reference]			
100-399	12.03 (10.18-14.23)^b^	<.001	3.30 (2.73-3.98)^b^	<.001
≥400	29.12 (24.46-34.67)^b^	<.001	4.52 (3.67-5.58)^b^	<.001
Teaching hospital	4.07 (3.72-4.45)^b^	<.001	1.40 (1.28-1.54)^b^	<.001
Cardiac capacity	8.04 (7.06-9.15)^b^	<.001	1.62 (1.40-1.87)^b^	<.001
Hospital is part of a system	2.19 (1.97-2.43)^b^	<.001	1.23 (1.11-1.36)^b^	<.001
Case mix index	6.81 (5.17-8.96)^b^	<.001	1.69 (1.36-2.09)^b^	<.001
**Community Characteristics**				
Stroke belt states	0.55 (0.46-0.66)^b^	<.001	0.71 (0.60-0.83)^b^	<.001
Another stroke center within 24-km radius	3.30 (2.96-3.68)^b^	<.001	1.32 (1.21-1.45)^b^	<.001
Urban hospitals	12.79 (10.64-15.37)^b^	<.001	2.15 (1.75-2.64)^b^	<.001
Percentage of population in HSA aged ≥65 y	0.90 (0.89-0.92)^b^	<.001	1.03 (1.02-1.04)^b^	<.001
Total population in HSA (log transformed)	1.60 (1.51-1.70)^b^	<.001	1.06 (1.00-1.12)^c^	.04
Percentage of variance explained by model^d^	NA	NA	0.53	NA
No. of hospital year–quarters	104 079	NA	104 079	NA

Abbreviations: HR, hazard ratio; HSA, hospital service area; NA, not applicable.

^a^ Bonferroni-adjusted 2-sided *P* values for multiple comparisons.

^b^ *P* < .01.

^c^ *P* < .05.

^d^ Percentage of variance explained for the multivariate model is obtained using methods described in Royston and Sauerbrei.^29^

For-profit hospitals (HR, 0.79; 95% CI, 0.69-0.89) and government-run hospitals (HR, 0.34; 95% CI, 0.29-0.40) were less likely to be stroke certified compared with not-for-profit hospitals (Table 2 and Figure 3). Compared with hospitals with fewer than 100 beds, the hazard rate of adopting stroke certification was 12.03 (95% CI, 10.18-14.23) times higher in hospitals with 100 to 399 beds and 29.12 (95% CI, 24.46-34.67) times higher in hospitals with 400 or more beds.

Geographically, urban hospitals were more likely to adopt stroke certification than rural hospitals (HR, 12.79; 95% CI, 10.64-15.37). Without controlling for other factors, hospitals in stroke belt states had a lower hazard rate of adopting stroke certification relative to hospitals in other states (HR, 0.55; 95% CI, 0.46-0.66).

### Multivariate Analyses

After controlling for other area and hospital characteristics, our multivariate results (Table 2, percentage of variance explained by model is 0.53)^29^ suggest that hospitals in more affluent HSAs continued to be more likely to adopt stroke certification. Specifically, the HR of hospitals in low-income HSAs to adopt stroke certification was 0.62 (95% CI, 0.52-0.74) and the HR of middle-income HSAs to adopt stroke certification was 0.78 (95% CI, 0.69-0.87) relative to hospitals in high-income HSAs. Hospitals in the lower tertile of profit-margin distribution were less likely to adopt stroke certification (HR, 0.87; 95% CI, 0.78-0.98) than hospitals in the upper tertile of profit-margin distribution. Geographically, we continued to find that rural hospitals and critical access hospitals had a substantially lower hazard rate of adopting stroke center certification. Larger hospitals also had a higher likelihood of adopting stroke certification, although at a lower magnitude than when the bivariate model was used (100-399 beds: HR, 3.3; 95% CI, 2.73-3.98; ≥400 beds: HR, 4.52; 95% CI, 3.67-5.58), as did teaching status, coronary artery bypass graft and percutaneous coronary intervention capability, system membership, not-for-profit status, and hospitals with a higher case mix index.

Stratifying our sample by urbanicity yielded similar results, with the exception that income level was not a significant determinant of stroke center adoption among rural hospitals (eTable 1 in the Supplement). Determinants of stroke center adoption were similar when we stratified by a hospital’s cardiac technology capacity and by their system membership. Use of the federal poverty line instead of median household income in the HSA also generated nearly identical results (HR, 0.77; 95% CI, 0.69-0.86 for middle tertile; HR, 0.74; 95% CI, 0.65-0.84 for upper tertile) (eTable 2 in the Supplement).

## Discussion

Overall, our analysis on the increase in stroke-certified hospitals across the study period of January 1, 2009, to September 30, 2017, found that not only were factors such as bed capacity, case-mix index, urbanicity, and service-level intensity associated with the likelihood of adopting stroke certification, but other financial factors such as economic condition of the HSA and profit margin of the hospital were also associated with stroke center certification adoption. Even after controlling for hospital and community factors, hospitals in low-income HSAs and those earning low profit margins continued to be less likely to adopt stroke certification than the reference hospitals by a large margin. For example, where an HR of 1 would mean that hospitals in both communities were adopting stroke certification at the same rate at any time point (ie, they have the same hazard rate), our finding of an HR of 0.58 in low-income HSAs indicates that the hazard rate in low-income communities is slightly more than one-half of that in the high-income community. This empirical evidence strongly suggests that economic incentives may contribute to a hospital’s adoption of stroke certification.

There are several reasons why economic factors may play a role. First, hospitals are incentivized to strive for stroke certification when their clientele are ready and able to pay for such care services. This consideration is not unlike those of any other service providers who are willing to invest in additional features over “plain vanilla” equivalents if they are able to charge a premium for their services. Second, there may be a reverse association. People who are affluent may opt to move to HSAs with better access to public resources, such as education and health care services (ie, Tiebout sorting),^30^ thus increasing the area’s mean income. Regardless of the mechanism behind the association, what is clear is that low-income communities are at risk of lacking good access to quality stroke care, a finding that has also been shown in another analysis that used block groups rather than hospitals.^6^

Our results also illustrate that early adopters were better equipped, had a higher profit margin, and were located in more affluent neighborhoods than late adopters. This lends some credence to the hypothesis that stroke certification could be part of a “medical arms race” among hospitals^31,32,33^ and that this increase in stroke-certified hospitals is affected by market signaling. Hospitals that have the best resources are the first to adopt stroke certification so as to disclose their quality to potential patients. Once that happens, the next-best hospitals (ie, late adopters) would be incentivized to adopt stroke certification. Other hospitals may choose not to pursue stroke center certification because some patients who are less information savvy may not perceive nondisclosure as a signal of low quality.

These reasons could help explain why certain hospitals, such as government hospitals, for example, were found to have a lower likelihood of adopting stroke certification. Evidence suggests that government hospitals are “caregivers of last resort” and provide unprofitable services that are in disproportionate demand from low-income and poorly insured patients.^34^ In these cases, government hospitals may be focusing any existing revenue into provision of safety-net services rather than more profitable services.^35^

We did not find that income variables were related to stroke center certification in rural areas. This is likely because rural areas are more uniformly low income: in our data, both the mean of rural median family income and the SD were notably lower than those of urban areas (eTable 1 in the Supplement). In addition, rural areas likely do not possess the capabilities required of a certified stroke center, including around-the-clock acute stroke team availability and availability of neurosurgical services within a certain time frame. Although volume is not a criterion for stroke certification status, the typically low volumes of a rural hospital would likely not support the need for such services, which have been shown to produce better outcomes with higher volumes.^36,37,38^

These findings have implications for population health. It is well known that vulnerable groups, such as uninsured and low-income patients, have a lower likelihood of receiving necessary treatment—whether intravenous thrombolytic procedures^20^ or endovascular treatment^14,16^—and have higher mortality rates.^15,22,39^ Our findings provide some evidence that the reason for these health disparities could be at the systems level and not merely attributable to other factors, such as a higher baseline incidence of stroke in certain populations.^40,41,42^ Given our consistent finding that adoption of stroke center certification is concentrated in high-income communities, further proliferation of stroke certifications may represent duplicative care. Given the well-documented association between volume and outcome in many conditions,^43,44,45^ including stroke,^36,37,38^ duplicative services and clustering^7^ in a geographic area could suppress the fixed volume of stroke treated in each center and worsen, rather than improve, quality of care. In fact, recent data suggest that, possibly owing to economic incentives, thrombectomy procedures have been shifting away from high-volume hospitals, potentially at the cost of quality and outcomes.^46^ The February 2018 passage of the Furthering Access to Stroke Telemedicine (FAST) Act, which mandates Medicare reimbursement for telestroke services regardless of where the patient receives treatment,^47^ may be one tool to ensure access. However, without additional mechanisms in place to prevent further expansion of stroke centers in redundant areas, the risk of diluting volume and potentially decreasing quality may remain.

On an even broader scale, our findings should be seen in the context of evidence demonstrating strong influence of market forces, which has also been found in the provision of other health care services, including emergency care,^48^ trauma care,^49,50,51^ and percutaneous coronary intervention.^52^ These findings may point to a need for a more rational system of allocation of health care services on a population level, such as incorporation of volume of certain conditions or procedures that qualify for certified stroke center designation, or regulation using a combination of disease incidence and geography.

### Limitations

There are several limitations to our study. First, accreditation bodies vary in their methods for granting approval to hospitals that apply for stroke center status, and there are some indications that not all stroke centers across these accreditations achieve similar performance.^53^ However, we also performed this analysis using data from the Joint Commission only and found nearly identical results. Second, economic and financial data tend to contain noise, which can cause attenuation bias in our estimates. This makes our estimate a lower bound. Third, the decision to seek stroke center certification can be affected by other unobservable factors, such as political considerations, underlying hospital culture, strategic maneuver, or baseline volume of patients with stroke in the community. Although some of the variables included in our model might represent noisy proxy of these unobserved characteristics, we cannot completely eliminate omitted variable bias. Finally, it is important to acknowledge that our analysis examines factors that are associated with adoption of stroke center certification and does not address the quality of stroke care in these hospitals.

## Conclusions

Our findings demonstrate the strong relationship between economic incentives—specifically, profit margin and income of the HSA—and a hospital’s decision to pursue stroke certification. We provide evidence that proliferation of stroke centers is uneven across geographic localities with a potential redundancy in certain affluent communities. Our results have important implications for the quality of care for patients with stroke and for population health and suggest that policymakers should consider taking a more active role in optimizing the locations of stroke-certified hospitals. The market-driven mechanism of allocating the supply of these essential health care services may be not only inefficient, but also potentially deleterious for vulnerable and nonvulnerable communities alike.

## Notes

Supplement. **eTable1.** Stratified Multivariate Hazard Ratios of Hospitals Achieving Stroke Certification by Urban/Rural Status, 2009 to 2017 **eTable2.** Sensitivity Analysis Using Poverty Line
