# Supplementary material for: Community and Hospital Factors Associated With Stroke Center Certification in the United States, 2009 to 2017
Source: JAMA Netw Open. 2019 Jul 26;2(7):e197855. doi: 10.1001/jamanetworkopen.2019.7855 (PMC6661722; doi:10.1001/jamanetworkopen.2019.7855)
Supplement: Supplement. — eTable1. Stratified Multivariate Hazard Ratios of Hospitals Achieving Stroke Certification by Urban/Rural Status, 2009 to 2017 eTable2. Sensitivity Analysis Using Poverty Line [file jamanetwopen-2-e197855-s001.pdf]

# Supplementary Online Content

Shen Y-C, Chen G, Hsia RY. Community and hospital factors associated with stroke center certification in the United States, 2009 to 2017. *JAMA Netw Open*. 2019;2(7):e197855. doi:10.1001/jamanetworkopen.2019.7855

**eTable1.** Stratified Multivariate Hazard Ratios of Hospitals Achieving Stroke Certification by Urban/Rural Status, 2009 to 2017

**eTable2.** Sensitivity Analysis Using Poverty Line

This supplementary material has been provided by the authors to give readers additional information about their work.

© Shen Y-C et al. *JAMA Network Open.*

## eTable 1. Stratified Multivariate Hazard Ratios of Hospitals Achieving Stroke Certification by Urban/Rural Status, 2009 to 2017

| | Rural Hospitals | | Urban Hospitals | | No Cardiac Tech Capacity | | Has Cardiac Tech Capacity | | Independent Hospitals | | Member of a System | |
|---|---|---|---|---|---|---|---|---|---|---|---|---|
| | **Hazard ratio** | **95% CI** | **Hazard ratio** | **95% CI** | **Hazard ratio** | **95% CI** | **Hazard ratio** | **95% CI** | **Hazard ratio** | **95% CI** | **Hazard ratio** | **95% CI** |
| **Economic and financial characteristics** | | | | | | | | | | | | |
| HSA family income (ref. grp. = Upper 1/3) | | | | | | | | | | | | |
| Middle income | 0.86 | [0.43,1.71] | 0.77** | [0.68,0.86] | 0.61** | [0.46,0.80] | 0.83** | [0.74,0.93] | 0.69** | [0.58,0.83] | 0.83** | [0.72,0.95] |
| Low income | 0.52 | [0.26,1.05] | 0.65** | [0.54,0.77] | 0.49** | [0.34,0.72] | 0.67** | [0.56,0.80] | 0.50** | [0.38,0.67] | 0.69** | [0.57,0.84] |
| Profit Margin (ref. grp. = Upper 1/3) | | | | | | | | | | | | |
| Middle 1/3 | 0.84 | [0.55,1.29] | 0.96 | [0.88,1.06] | 1.32* | [1.02,1.71] | 0.90* | [0.81,0.99] | 1.05 | [0.86,1.27] | 0.93 | [0.84,1.04] |
| Lower 1/3 | 0.95 | [0.58,1.54] | 0.86* | [0.77,0.97] | 1.15 | [0.88,1.51] | 0.83** | [0.73,0.94] | 0.99 | [0.79,1.25] | 0.85* | [0.75,0.97] |
| **Hospital Characteristics** | | | | | | | | | | | | |
| Ownership (ref. grp. = Not-for-profit) | | | | | | | | | | | | |
| For-profit | 0.90 | [0.53,1.52] | 0.89 | [0.79,1.00] | 0.86 | [0.66,1.13] | 0.88 | [0.78,1.00] | 0.40** | [0.21,0.77] | 0.92 | [0.81,1.03] |
| Government | 1.03 | [0.58,1.85] | 0.85* | [0.72,0.99] | 0.70 | [0.47,1.04] | 0.87 | [0.74,1.03] | 0.83 | [0.68,1.01] | 0.76* | [0.60,0.97] |
| Designated critical access hospital | 0.11** | [0.05,0.24] | 0.16** | [0.06,0.43] | 0.14** | [0.08,0.27] | 0.00 | [0.00,0.00] | 0.20** | [0.09,0.43] | 0.08** | [0.03,0.19] |
| Total Hospital Beds (ref. grp. = <100 beds) | | | | | | | | | | | | |
| 100-399 beds | 2.77** | [1.71,4.51] | 3.28** | [2.67,4.02] | 3.30** | [2.56,4.27] | 2.60** | [1.99,3.39] | 3.43** | [2.33,5.06] | 2.95** | [2.39,3.64] |
| 400+ beds | 5.89** | [2.87,12.08] | 4.61** | [3.70,5.75] | 6.59** | [4.45,9.76] | 3.61** | [2.72,4.78] | 5.04** | [3.27,7.77] | 3.94** | [3.11,5.00] |
| Teaching hospitals | 1.73* | [1.10,2.71] | 1.39** | [1.26,1.53] | 1.67** | [1.32,2.12] | 1.34** | [1.21,1.48] | 1.50** | [1.25,1.80] | 1.35** | [1.20,1.51] |
| Has cardiac capacity (either cath. lab or CABG) | 1.87* | [1.13,3.11] | 1.55** | [1.34,1.81] | | | | | 2.06** | [1.52,2.79] | 1.44** | [1.22,1.69] |
| Hospital is part of a system | 1.37 | [0.93,2.02] | 1.23** | [1.11,1.36] | 1.94** | [1.50,2.50] | 1.09 | [0.98,1.21] | | | | |
| Case mix index | 6.85** | [2.98,15.71] | 1.54** | [1.25,1.91] | 1.86** | [1.23,2.83] | 1.61** | [1.25,2.09] | 1.97** | [1.34,2.92] | 1.74** | [1.35,2.25] |
| **Community Characteristics** | | | | | | | | | | | | |
| Stroke belt states | 0.79 | [0.48,1.30] | 0.71** | [0.60,0.84] | 0.51** | [0.32,0.80] | 0.74** | [0.62,0.87] | 0.78 | [0.59,1.03] | 0.67** | [0.55,0.82] |
| Has another stroke center within 15-mile radius | 1.39 | [0.60,3.21] | 1.32** | [1.21,1.45] | 1.34* | [1.04,1.73] | 1.32** | [1.20,1.45] | 1.27** | [1.07,1.51] | 1.35** | [1.21,1.50] |
| Percent population in HSA that are >=65 years old | 1.08** | [1.04,1.13] | 1.03** | [1.02,1.04] | 1.09** | [1.06,1.12] | 1.02** | [1.01,1.03] | 1.03* | [1.00,1.05] | 1.03** | [1.02,1.05] |
| Total population in HSA (log transformed) | 1.27* | [1.04,1.55] | 1.05 | [0.99,1.10] | 1.04 | [0.94,1.16] | 1.08** | [1.03,1.14] | 1.01 | [0.92,1.10] | 1.09** | [1.03,1.15] |
| Urban hospitals | | | | | 2.72** | [1.88,3.94] | 1.80** | [1.41,2.29] | 2.10** | [1.48,2.98] | 2.11** | [1.64,2.72] |
| N | 60852 | | 43227 | | 77865 | | 26214 | | 48583 | | 55496 | |

© Shen Y-C et al. *JAMA Network Open.*

**eTable 2. Sensitivity Analysis Using Poverty Line**

| | Hazard ratio | 95% CI | Bonferroni-adjusted p-values |
|---|---|---|---|
| **Economic and financial characteristics** | | | |
| HSA poverty level (ref. grp. = Lower 1/3) | | | |
| Middle 1/3 | 0.77** | [0.69,0.86] | <0.001 |
| Upper 1/3 | 0.74** | [0.65,0.84] | <0.001 |
| Profit Margin (ref. grp. = Upper 1/3) | | | |
| Middle 1/3 | 0.96 | [0.87,1.06] | 0.829 |
| Lower 1/3 | 0.88* | [0.78,0.98] | 0.049 |
| **Hospital Characteristics** | | | |
| Ownership (ref. grp. = Not-for-profit) | | | |
| For-profit | 0.86* | [0.77,0.97] | 0.021 |
| Government | 0.81** | [0.70,0.95] | 0.017 |
| Designated critical access hospital | 0.13** | [0.07,0.23] | <0.001 |
| Total Hospital Beds (ref. grp. = <100 beds) | | | |
| 100-399 beds | 3.20** | [2.65,3.87] | <0.001 |
| 400+ beds | 4.41** | [3.58,5.43] | <0.001 |
| Teaching hospitals | 1.40** | [1.28,1.54] | <0.001 |
| Has cardiac capacity (either cath. lab or CABG) | 1.61** | [1.39,1.87] | <0.001 |
| Hospital is part of a system | 1.23** | [1.12,1.37] | <0.001 |
| Case mix index | 1.66** | [1.34,2.05] | <0.001 |
| **Community Characteristics** | | | |
| Stroke belt states | 0.67** | [0.57,0.79] | <0.001 |
| Has another stroke center within 15-mile radius | 1.32** | [1.20,1.45] | <0.001 |
| Percent population in HSA that are >=65 years old | 1.03** | [1.02,1.04] | <0.001 |
| Total population in HSA (log transformed) | 1.13** | [1.07,1.18] | <0.001 |
| Urban hospitals | 2.26** | [1.85,2.77] | <0.001 |
| N | 104079 | | |

© Shen Y-C et al. *JAMA Network Open.*
